# [Expression of Concern] Fasudil, a Rho-associated protein kinase inhibitor, attenuates retinal ischemia and reperfusion injury in rats

**DOI:** 10.3892/ijmm.2026.5803

**Published:** 2026-03-18

**Authors:** Haishan Song, Dianwen Gao

Int J Mol Med 28: 193-198, 2011; DOI: 10.3892/ijmm.2011.659

Following the publication of the above paper, it was drawn to the Editor's attention by a concerned reader that, regarding the photomicrographs shown in Fig. 1 on p. 195, the 'I/R' and 'Control' data panels (for the Day 1 experiments) contained an overlapping section, such that data which were intended to show the results of differently performed experiments were apparently derived from the same original source.

The authors have been contacted by the Editorial Office to offer an explanation for the apparent anomaly in the presentation of the data in their paper, and we are awaiting their response. Owing to the fact that the Editorial Office has been made aware of potential issues surrounding the scientific integrity of this paper, we are issuing an Expression of Concern to notify readers of this potential problem while the Editorial Office continues to investigate this matter further.

